# Medical Treatment of Disordered Menstruation

**Published:** 1905-10

**Authors:** 


### ABSTRACTS.

### Medical Treatment of Disordered Menstruation.

Geo. S. Walker, First Assistant Physician in charge of female department, Western State Hospital, Staunton, Va., *(Brooklyn Medical Journal)* remarks that he was looking for a safe and efficient emmenagogue, which gave positive results in cases of amenorrhea, dysmenorrhea and suppressed menstruation, without either exciting or depressing the patient; without causing any disturbance on the part of the digestive tract or the urinary tract, such as are met with the use of most remedies classed as emmenagogues. He knew that apiol was a substance that had long been known to possess marked emmenagogue properties, but that it had not been used extensively in this country on account of certain unpleasant after-effects. The ordinary apiol of commerce it seemed, was simply a mixture of impure principles obtained from parsley by extraction. He determined, therefore, to try ergo apiol by Martin H. Smith Company, New York, a liquid substance dispensed in gelatine capsules which contains the pure apiol in addition to a combination of emmenagogues that immediately appealed to him as calculating to enhance the efficacy of the whole remedy. He selected a series of cases in the hospital, each of which was chacterised by a more or less pronounced menstrual disorder of some standing, and administered no other medication for the disordered menstruation than ergo apiol (Smith). He cites in illustration, three cases in which the remedy in question was employed. They are only examples of the experience he had with it.

Case I. Age twenty-one. Said she had not menstruated for nearly a year and attributed her suffering in body and mind to this fact. She was depressed and on the verge of committing suicide. I began to give her two capsules of ergo apiol three times a day until after her second period. She improved both mentally and physicially during the time of taking this remedy and her condition was so remarkably ameliorated that she was discharged cured when she menstrual function had been re-established.

Case II. Aged twenty-four years. Has been suffering from amenorrhea for a year which persisted in spite of all treatment. She was melancholy and had a very poor appetite and other disturbances due to suppressed menstruation. I began giving her two capsules of ergo apiol twice a day. Finally her menses returned. The menstruation was perfectly normal. Since the re-establishment of her normal function the patient has gained both mentally and physicially and regained her mental balance and her usual cheerfulness so that she was discharged cured.

From my experience with ergo apiol (Smith) and from the experiences of a number of other observers, whose findings are published in the literature of the past few years, this remedy represents an emmenagogue of the highest type of efficiency, combined with the inestimable advantages of safety, trustworthiness and absence of any unpleasant after-effects.

